# Inhibition of Transglutaminase 2 as a Potential Host-Directed Therapy Against *Mycobacterium tuberculosis*

**DOI:** 10.3389/fimmu.2019.03042

**Published:** 2020-01-24

**Authors:** Ivana Palucci, Giuseppe Maulucci, Flavio De Maio, Michela Sali, Alessandra Romagnoli, Linda Petrone, Gian Maria Fimia, Maurizio Sanguinetti, Delia Goletti, Marco De Spirito, Mauro Piacentini, Giovanni Delogu

**Affiliations:** ^1^Fondazione Policlinico Universitario A. Gemelli IRCCS, Rome, Italy; ^2^Institute of Microbiology, Università Cattolica del Sacro Cuore, Rome, Italy; ^3^Institute of Physics, Università Cattolica del Sacro Cuore, Rome, Italy; ^4^Electron Microscopy and Cell Biology Unit, Department of Epidemiology and Preclinical Research, “L. Spallanzani” National Institute for Infectious Diseases (INMI), IRCCS, Rome, Italy; ^5^Translational Research Unit, Department of Epidemiology and Preclinical Research, “L. Spallanzani” National Institute for Infectious Diseases (INMI), IRCCS, Rome, Italy; ^6^Department of Molecular Medicine, Sapienza University of Rome, Rome, Italy; ^7^Department of Biology, University of Rome “Tor Vergata”, Rome, Italy; ^8^Mater Olbia Hospital, Olbia, Italy

**Keywords:** tuberculosis, transglutaminase 2, host-directed therapy, *Mycobacterium tuberculosis*, macrophage, MDR-TB

## Abstract

Host-directed therapies (HDTs) are emerging as a potential valid support in the treatment of drug-resistant tuberculosis (TB). Following our recent report indicating that genetic and pharmacological inhibition of transglutaminase 2 (TG2) restricts *Mycobacterium tuberculosis* (*Mtb*) replication in macrophages, we aimed to investigate the potentials of the TG2 inhibitors cystamine and cysteamine as HDTs against TB. We showed that both cysteamine and cystamine restricted *Mtb* replication in infected macrophages when provided at equimolar concentrations and did not exert any antibacterial activity when administered directly on *Mtb* cultures. Interestingly, infection of differentiated THP-1 mRFP-GFP-LC3B cells followed by the determination of the autophagic intermediates pH distribution (AIPD) showed that cystamine inhibited the autophagic flux while restricting *Mtb* replication. Moreover, both cystamine and cysteamine had a similar antimicrobial activity in primary macrophages infected with a panel of *Mtb* clinical strains belonging to different phylogeographic lineages. Evaluation of cysteamine and cystamine activity in the human *ex vivo* model of granuloma-like structures (GLS) further confirmed the ability of these drugs to restrict *Mtb* replication and to reduce the size of GLS. The antimicrobial activity of the TG2 inhibitors synergized with a second-line anti-TB drug as amikacin in human monocyte-derived macrophages and in the GLS model. Overall, the results of this study support the potential usefulness of the TG2-inhibitors cysteamine and cystamine as HDTs against TB.

## Introduction

Tuberculosis (TB) is a leading cause of death worldwide with 10 million new TB cases and 1.6 million deaths in 2017 alone (1). The emergence and spread of *Mycobacterium tuberculosis* (Mtb) strains resistant to the two most common drugs isoniazid and rifampicin (multidrug-resistant Mtb, MDR-TB) are a cause of major concern. Among the half million cases of MDR-TB estimated in 2017, 8.5% are expected to have a pattern of extensively drug resistant-TB (XDR-TB), defined as the additional non-susceptibility to fluoroquinolones and an injectable drug (1). Drug regimens for MDR-TB patients are much more complex and toxic compared to those commonly administered to patients with drug-susceptible TB and consist in the combined administration of at least four drugs for up to 20 months (2, 3). Despite the introduction of new drugs, therapeutic regimens of MDR-TB and XDR-TB patients show poor success rates that rarely exceed 50% in high-burden countries (4). Moreover, these regimens are very expensive; combining direct and indirect costs, in EU states and the US, the average cost for an MDR-TB patient is five to six times higher than a drug-susceptible patient and increases up to 20 times for XDR-TB (2, 5). These high costs associated with the treatment of drug-resistant TB pose a major burden to many countries, with relevant health, social, and economic consequences (2).

There is an urgent need of improved treatment options for TB, and the introduction of the new drugs delamanid and bedaquiline, while widening the therapeutic options, has already led to the emergence of *Mtb* strains resistant to these drugs (6), frustrating the hopes of scientists, public health authorities, and patients. In the last few years, also thanks to new insights in TB pathogenesis, several host-directed therapies (HDTs) have been proposed as adjunct therapy against TB and primarily against the drug-resistant forms that do not respond to the available treatments (7–9). Some of these HDTs are based on the repurposing of old drugs which have already shown a good safety record in previous clinical trials (7, 8), as is the case for metformin (10), statins (11), and other drugs (12). These treatments may enhance the host antimicrobial defenses or provide beneficial effects by interfering with the mechanisms exploited by the pathogen to persist in host tissues or by lessening inflammation and reducing tissue damage. These beneficial effects of HDTs can synergize with the anti-TB regimens, resulting in improved clinical outcomes and reduced risk for emergence of drug resistance, and may lead to shorter anti-TB regimens.

Transglutaminase 2 (TG2) is a pleiotropic enzyme belonging to the transglutaminase family involved in several important cellular processes including cell death/survival and autophagy (13–15). We have recently shown that genetic or pharmacological inactivation of TG2 enhances the anti-mycobacterial properties of *Mtb*-infected macrophages, which intriguingly correlate with reduced cell death and impairment of the LC3/autophagy homeostasis (16). Interestingly, two TG2 inhibitors, cystamine and cysteamine, have already been tested in clinical trials and showed a good safety record (17, 18). Briefly, cystamine inhibits most of the extracellular transglutaminases, while its reduced form cysteamine can more efficiently reach the cytoplasm and inhibit transglutaminase intracellular activities (19). In this study, we aimed to investigate in relevant *in vitro* and *ex vivo* models of human *Mtb* infection whether these two TG2 inhibitors act as HDTs against TB.

## Results

### Cysteamine and Cystamine Act as a Host-Directed Therapy Against *Mtb*

**Figure 1:**
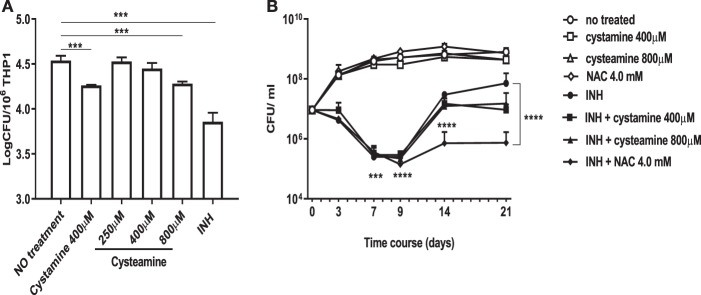
Cystamine and cysteamine exert anti-mycobacterial activity only in infected macrophages. **(A)** THP-1 cells were infected with *Mtb* at MOI 1:10 and treated with the drugs starting at 4 h p.i. until harvesting of intracellular CFU at 2 days p.i. Cystamine (400 μM) or cysteamine at different concentrations (from 250 to 800 μM) and isoniazid at MIC concentration were administered to infected macrophages 4 h p.i. The graph shows intracellular CFU at 2 days p.i. for the untreated and treated THP-1. Data were analyzed by one-way ANOVA followed by Dunnett's multiple-comparisons test (***p < 0.005, compared with *Mtb* H37Rv no treatment). **(B)** Viability of *Mtb* treated with INH (7.3 μM, 20 times the MIC), cystamine (400 μM) or cysteamine (800 μM), NAC (4.0 mM), and combination of these with INH. The experiments, in which the combinations are shown, were performed using the same concentrations of drugs as the individual treatments. Aliquots were taken at indicated times and plated to determine CFU. Average with SD is plotted (*n* = 1). Values are expressed as a mean of three independent experiments. Data were analyzed by one-way ANOVA with Dunnett's multiple-comparisons test against *Mtb* H37Rv untreated (***p < 0.005, ****p < 0.001).

We have recently shown that treatment of murine and human primary macrophages with cystamine, a TG2 inhibitor, enhances the anti-tuberculosis activity of macrophages (16). The reduced form of cystamine, cysteamine, is an orphan drug also well-known as TG2 inhibitor already tested in clinical studies to treat non-infectious diseases (18). To investigate whether cysteamine had an anti-microbial activity against *Mtb* in macrophages, THP-1 monocyte-derived macrophages were infected with *Mtb* H37Rv and then treated with cystamine and cysteamine at concentrations compatible to those achieved *in vivo* (16). As shown in Figure 1A, treatment with cysteamine resulted in a dose-dependent reduction of intracellular bacteria that reached a similar activity with cystamine when administered at equimolar concentrations (400 μM cystamine, 800 μM cysteamine). At these concentrations, treatment with cystamine or cysteamine did not reduce macrophage cell viability (as assessed by measuring lactate dehydrogenase, data not shown) nor inhibit *Mtb* H37Rv viability in axenic culture (Figure 1B), similar to what was previously shown for cystine or cysteine (20). Moreover, the combined use of isoniazid with these two drugs, at concentrations previously used in macrophages, provided only a slight delay in the emergence of drug-resistant bacteria. Besides, these treatments did not result in the sterilization or strong inhibition of the persistent population (Figure 1B), as previously observed with other molecules with a free-thiol group [though when administered at higher concentration as is the case of N-acetylcysteine (NAC) at 4 mM; Figure 1B] (20). Taken together these results indicate that cystamine and cysteamine, at the concentrations shown to inhibit *Mtb* replication in macrophages, do not exert any direct antimicrobial effect on *Mtb*.

### Cystamine Restricts *Mtb* Replication in Macrophages While Inhibiting Autophagy

We previously showed that genetic inactivation of TG2 in murine macrophages results in the impairment of the LC3/autophagy homeostasis, which nevertheless correlates with the restriction of *Mtb* intracellular replication (16). To further investigate the impact of the two TG2 inhibitors cystamine and cysteamine on autophagy, we quantitatively evaluated the autophagic flux by confocal pH-imaging of the autophagic intermediates on THP-1 cells transfected with mRFP-GFP-LC3B (21). The number and pH of autophagic intermediates are expressed by autophagic intermediates pH distribution (AIPD), the pH distribution of the number of autophagic intermediates per cell. AIPD shape and amplitude are sensitive to alterations in the autophagy pathway induced by drugs or environmental states and allow a quantitative estimation of autophagic flux by retrieving the concentrations of autophagic intermediates. Briefly, the total area of the AIPD corresponds to the total number of autophagic intermediates. An increase of high *F*_G_/*F*_R_ organelles indicates an increase of autophagomes. Formation of autolysosomes (autophagosome–lysosome fusion) is indicated by a shift of AIPD toward low *F*_G_/*F*_R_ values, caused by a decrease of the pH of autophagic intermediates. Thus, this assay is not only a marker of autophagy activation but also allows for an accurate estimation of the autophagic flux (21).

**Figure 2:**
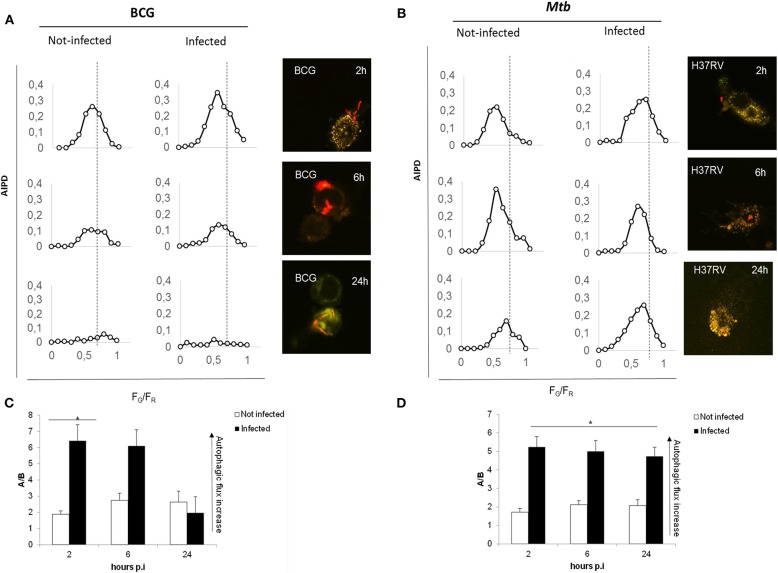
Autophagic flux is altered in virulent *Mtb* H37Rv strain compared to *M. bovis* BCG. THP-1 RFP-LC3-GFP cells infected with *M. bovis* BCG Ds-Red Cherry at MOI 5:1 **(A)** and with virulent *Mtb* H37Rv Ds-Red Cherry at MOI 1:1 **(B)**. Quantitative assessment of autophagic flux during the time interval lasting 24 h post-infection. Reported in the graphs are the number and pH of autophagic intermediates expressed by AIPD, the pH distribution of the number of autophagic intermediates per cell. AIPD shape and amplitude are sensitive to alterations in the autophagy pathway induced by drugs or environmental states and allow a quantitative estimation of autophagic flux by retrieving the concentrations of autophagic intermediates. An increase of high *F*_G_/*F*_R_ organelles indicates an increase of autophagomes. A shift of AIPD toward low *F*_G_/*F*_R_ values indicates that the pH of autophagic intermediates is shifting to acidic values and autolysosomes formation. **(C,D)** *Bar graphs* on the trend of autophagy during infection of *M. bovis* BCG and *Mtb*, respectively, expressed as ratios *F*_G_/*F*_R_ (*A*/*B*) between the AIPD area of a fixed threshold value. Data were analyzed by two-way ANOVA with Bonferroni's multiple-comparisons test (*p < 0.05).

We first assessed the suitability of the assay following infection with the virulent *Mtb* H37Rv and the attenuated strain *Mycobacterium bovis* BCG, which is unable to inhibit autophagy and is readily degraded by macrophages (22). Infection with *Mtb* and BCG expressing the Ds-Red Cherry fluorescent protein, followed by the confocal analysis of autophagic intermediates (21), allows distinguishing autophagic activation and flux in infected and non-infected cells (Figure 2). A visual inspection of the AIPDs reveals that, in BCG-infected cells (Figure 2A), not only autophagosomes are formed at 2 h post-infection (p.i.) (increase of high *F*_G_/*F*_R_ shoulder) but also autolysosomes are forming (simultaneous shift of AIPD toward low *F*_G_/*F*_R_ values). The observed decrease of the total number of intermediates during the time course indicates an increased autophagic flux accompanied by a gradual autophagy inactivation (intermediates almost disappear at 24 h). Therefore, this indicates that the overall duration of the autophagy process in THP-1 cells infected with BCG is ≈24 h (Figures 2A–C).

Infection with virulent *Mtb* (Figures 2B–D) activates autophagy, though the AIPD shift toward acidic pH is less pronounced compared to BCG-infected cells and is accompanied by an increase of the neutral organelles. In contrast with the correspondent non-infected cells, the peak of autophagosomes (high *F*_G_/*F*_R_ values) is higher than the peak of autolysosomes (low *F*_G_/*F*_R_ values; Figure 2B). This change in the shape of the distribution indicates *Mtb* inhibition of the autophagic flux following infection by preventing intermediate acidification, in line with previous findings (22–24). Another important difference between BCG- and *Mtb*-infected macrophages is that AIPD in the latter does not undergo important changes in shape over the same 24-h time course, indicating that cells keep autophagy activated even at 24 h p.i. These results underscore the usefulness of the quantitative analysis of AIPD to monitor authophagy in macrophages infected with *Mtb*. Of note, we also observed autophagic flux induction in non-infected macrophages (in BCG- and *Mtb*-infected cells), probably resulting from the cytokines released by infected cells (24, 25) that can act in paracrine mode.

**Figure 3:**
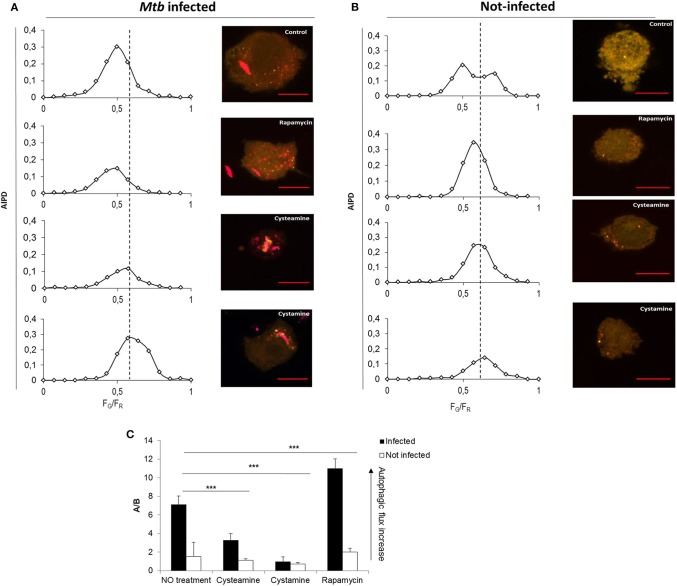
Cystamine and cysteamine block autophagy in *Mtb*-infected macrophages. Representative confocal laser scanning images of THP-1 RFP-LC3-GFP infected with *Mtb* H37Rv Ds-Red at MOI 1:1. At 4 h p.i., cystamine (400 μM), cysteamine (800 μM), or rapamycin (1 mM) was added, and the images were acquired at 24 h p.i. The images represent the merge of the green and the red channels. Autophagic intermediates pH distribution (average of *n* = 80 cells) for the same treatments is reported along the correspondent representative images. **(C)** Histogram of the ratios *F*_G_/*F*_R_ (*A*/*B*) between the AIPD area at the left **(A)** and at the right **(B)** of a fixed threshold value. An increase in *A*/*B* value corresponds to an increase in the autophagic flux. Data were analyzed by two-way ANOVA with Bonferroni's multiple-comparisons test (***p < 0.005).

To investigate the impact of the TG2 inhibitors on autophagy, THP-1 mRFP-GFP-LC3B cells were infected with *Mtb* H37Rv Ds-Red Cherry and then treated with rapamycin, cystamine, and cysteamine immediately after infection, and AIPD was measured at 24 h later (Figure 3). As expected, treatment with rapamycin readily induced an increase in autophagic flux; AIPD displays an acidification of intermediates (Figure 3A) with respect to untreated *Mtb*-infected macrophages (Figure 3B). Conversely, treatment with cysteamine resulted in a decrease of autophagosome acidification (Figures 3A,B), thus indicating a partial inhibition of the autophagic flux at the level of autophagosome maturation. To quantify the extent of the activation or inhibition of the autophagic flux, we reported in Figure 3C the ratio *A*/*B* between the AIPD area at the left (*A*) and at the right (*B*) of a fixed threshold value (*F*_G_/*F*_R_ = 0.55). An increase in *A*/*B* value corresponds to an increase in the autophagic flux. These findings are in full agreement with the impairment of late autophagic stages reported in TG2 knockout mice (26). Taken together, these results indicate that treatment with cystamine, and to a lesser extent cysteamine, of THP-1 mRFP-GFP-LC3B cells infected with *Mtb* results in the inhibition of the autophagic flux.

### Pharmacological Inhibition of TG2 Restricts *Mtb* Replication of Modern and Ancient *Mtb* Clinical Isolates

**Figure 4:**
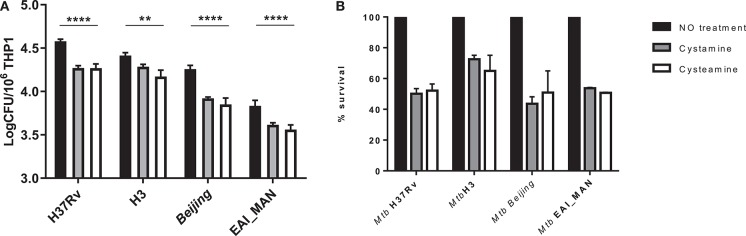
Evaluation of cystamine and cysteamine effects in macrophages infected with different *Mtb* strains belonging to different lineages. Differentiated THP-1 cells were infected with MTBC clinical strains belonging to different clades (H3, Beijing, EAI_MAN) (25) at MOI 1:1. At 4 h p.i., the cells were treated with cystamine (400 μM) and cysteamine (800 μM), and at 2 days p.i., cells were lysed to measure intracellular CFU **(A)**. Values are expressed as a mean of three independent experiments. **(B)** To compare the activity of the two drugs, results are expressed as percentage of mean value of CFU in triplicate of treated vs. untreated strains in panels. Data were analyzed by two-way ANOVA with Dunnett's multiple-comparisons test against each strain with untreated condition (**p < 0.01, ****p < 0.001).

*Mtb* strains belonging to different phylogeographic lineages show different pathogenetic properties, with implications in terms of virulence, extent of disease, transmission, and epidemic potentials (25, 27–29). *Mtb* strains belonging to modern lineages showed enhanced virulence compared with strains of the ancient lineages, and recent data from our group indicate a different ability to induce and evade autophagy by modern vs. ancient strains (25). To investigate whether treatment with TG2 inhibitors could restrict the intracellular replication of *Mtb* belonging to different lineages, THP-1 cells were infected with *Mtb* clinical isolates of the modern Euro-American (H3 clade) and East Asian (Beijing) lineages and of the ancient lineage EAI (EAI_MAN). As shown in Figure 4, treatment with cysteamine and cystamine were equally effective in restricting *Mtb* replication of strains of different clades, with a decrease over the untreated control that ranged between 35 and 50% (Figure 4B). Interestingly, we show a 50% decrease in THP-1 cells infected with the Beijing *Mtb* strain. These results indicate that cysteamine and cystamine promote an antimicrobial activity in macrophages effective against clinical isolates representative of the *Mtb* genetic diversity at global level.

### Cystamine Synergizes With Capreomycin in Restricting *Mtb* Replication in Primary Human Monocyte-Derived Macrophages

**Figure 5:**
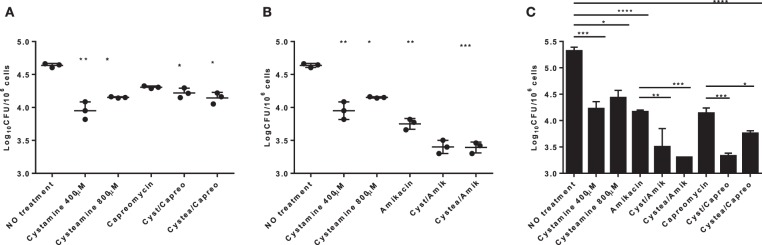
Evaluation of the synergistic effect of cystamine and cysteamine with aminoglycosides in human primary monocyte-derived macrophages (hMDM). hMDM were infected with *Mtb* H37Rv at MOI 1:1, and at 4 h p.i., we added different drugs: cystamine (400 μM), cysteamine (800 μM); the antibacterial drugs belonging to the aminoglycosides class capreomycin (4 μg/ml, **A**) and amikacin (1 μg/ml, **B**) and the combination of the aminoglycosides with cystamine and cysteamine. Two days after infection, cells were lysed to assess intracellular CFU, and results are shown as log CFU/10^6^ cells. **(C)** To measure the long-term effect in this *in vitro* model of *Mtb* infection, hMDM were maintained up to 7 days p.i., and CFU were determined. Values are expressed as a mean of three independent experiments. Data were analyzed by one-way ANOVA followed by Dunnett's multiple-comparisons test (*p < 0.05, **p < 0.001, ***p < 0.005, ****p < 0.001 compared with *Mtb* H37Rv no treatment). To measure the synergistic effect of cystamine and cysteamine in combination with capreomycin or amikacin in prolonged treatment, we compared groups treated with antibiotic alone with those receiving the same antibiotic in combination with cysteamine or cystamine (**p < 0.01, ***p < 0.005 for amikacin treatments; *p < 0.05, ***p < 0.001 for capreomycin treatment). Data obtained from single independent infections are reported in Supplementary Figure 1.

HDTs against TB have the potential to synergize with antimicrobial drugs to enhance the efficacy of therapy. This is of utmost importance during treatment of drug-resistant TB, which relies on antibiotics that are less powerful than the first-line drugs (9). As a proof of concept, to investigate the potential usefulness of the TG2-inhibitors under study, human monocyte-derived macrophages (hMDM) were infected with *Mtb* and then treated with cystamine, cysteamine alone, or in combination with the second-line anti-TB drug capreomycin. As shown in Figure 5, cystamine reduced *Mtb* replication in macrophages at a higher level compared to rapamycin (16) and similarly to capreomycin when these drugs were administered at 4 h p.i. and intracellular *Mtb* evaluated after 2 days of infection. Interestingly, the combined use of cystamine and capreomycin further reduced *Mtb* replication in macrophages, indicating a synergistic effect of these drugs. A similar experiment was repeated with amikacin, an aminoglycoside included in group C of drugs endorsed for use in longer MDR-TB regimens (30). As shown in Figure 5B, in hMDM, amikacin significantly reduced *Mtb* intracellular growth even more than the reduction generated by capreomycin (capreomycin = −0.32 log colony-forming units (CFU)/10^6^ cells; amikacin = −0.89 log CFU/10^6^ cells). Remarkably, the combined use of amikacin and cysteamine or cystamine further reduced *Mtb* replication in hMDM, providing a decrease of −1.22 log CFU/10^6^ cells for combination with cystamine and −1.24 log CFU/10^6^ cells for cysteamine over untreated infected hMDM (Figure 5B). Interestingly, the respective anti-*Mtb* activity of amikacin and capreomycin was lower at day 7 p.i. compared to what was observed at day 2 p.i. (Figure 5C); differently, the combination of aminoglycosides, particularly amikacin, with cystamine and cysteamine resulted in a persistent and highly significant reduction of intracellular CFU (Figure 5C). Taken together, these results indicate that cystamine and cysteamine can synergize with amikacin to enhance anti-TB activity in infected hMDM.

### Cysteamine and Cystamine Are Active Against *Mtb* in the Human *ex vivo* Model of Granuloma-Like Structures

**Figure 6:**
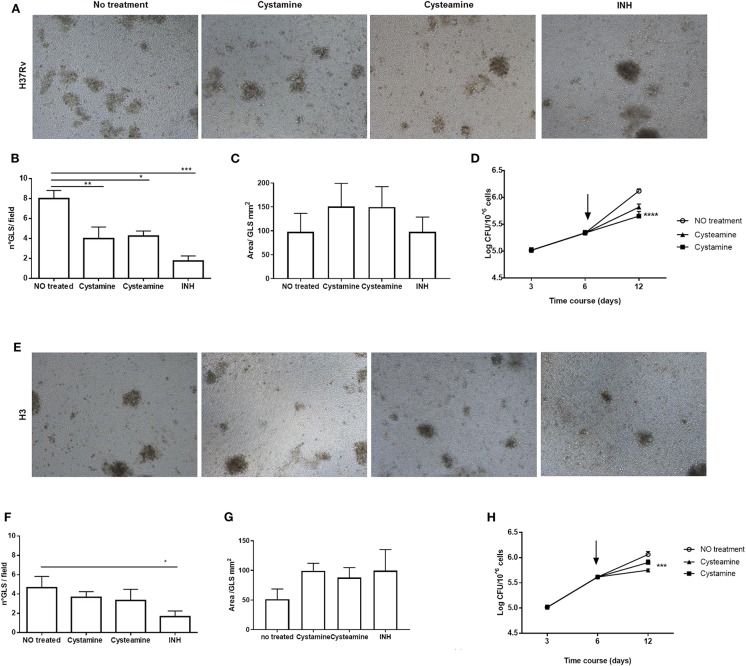
Cysteamine and cystamine reduce the fitness of *Mtb* in an *in vitro* granuloma model containing human innate and adaptive immune cells. PBMCs obtained from healthy donors were infected with *Mtb* H37Rv and *Mtb* H3 (MOI 1:1) for up to 12 days. Representative images of granuloma-like structure formed *in vitro* 10 days after infection with *Mtb* reference strain H37Rv **(A)** and H3 clinical strain **(E)**. Magnification, ×40. Granuloma formation was scored for each condition; the means ± standard deviations of scores representative of three experiments each are given in the images. GLS were treated with different drugs at 3 days post-infection; the medium was replaced: cystamine (400 μM), cysteamine (800 μM), and INH at MIC concentration. The measurement of the number of GLS and area was applied on day 10 p.i., 12 fields per sample were evaluated (**B,C** for *Mtb* H37Rv; **F,G** for *Mtb* H3), and CFU were determined at 3, 6, and 12 days post-infection **(D)** and for clinical strain *Mtb* H3 **(H)**, *arrows* represent the beginning of treatments (*p < 0.05, **p < 0.01, ***p < 0.005, ****p < 0.001).

Infection of human peripheral blood monocyte cells (PBMCs) with *Mtb* results in the formation of granuloma-like structures (GLS) that are emerging as a valuable *ex vivo* model of TB (31, 32). To investigate the activity of these HDTs against TB, PBMCs were infected with *Mtb* H37Rv and with the clinical strain *Mtb* H3, which in hMDM showed enhanced virulence compared with other *Mtb* reference and clinical strains (25). Following infection with *Mtb*, cysteamine, or cystamine was added in infected GLS at day 6 p.i. at the concentrations previously used in macrophages. At day 12 p.i., the total CFU counts were evaluated, and some GLS parameters were analyzed. As shown in Figures 6A–D, treatment with cysteamine and cystamine resulted in a reduction in the number of GLS per field compared with untreated GLS, while no differences were observed in the average surface area of these GLS. Interestingly, *Mtb* H37Rv load was significantly reduced in these GLS, confirming the anti-mycobacterial activity of these two TG2 inhibitors. Infection of PBMCs with *Mtb* H3 resulted in fewer GLS with smaller areas compared with the results obtained with *Mtb* H37Rv (Figures 6E–G). Again, cystamine significantly reduced the total CFU of *Mtb* H3-infected GLS, while the activity of cysteamine was lower compared with the results observed in *Mtb* H37Rv-infected GLS (Figure 6H). Taken together, these results indicate that cysteamine and cystamine reduce *Mtb* growth in the human *ex vivo* model of GLS.

**Figure 7:**
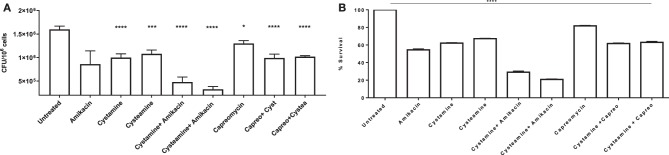
Synergy effect of cysteamine and cystamine with aminoglycosides in GLS model. Granuloma-like structures obtained from healthy donors were infected with *Mtb* H37Rv at MOI 1:1 and were treated with different drugs, at 3 days post-infection as described above, with the addition of co-treatment of cysteamine and cystamine with amikacin (1 μg/ml) and capreomycin (4 ug/ml). CFU were determined at 12 days post-infection **(A)**; *p < 0.05, ***p < 0.005, ****p < 0.001 by one-way ANOVA with Dunnett's multiple-comparisons test compared with *Mtb* H37Rv no treatment. To highlight the inhibition of mycobacterial survival following the addition of different drugs, we expressed the results as percentage of survival of treated vs. untreated cells in **B**.

To further assess the potentials of these two TG2 inhibitors as HDTs for TB, the respective activity of cystamine and cysteamine was assessed in combination with capreomycin and amikacin in the GLS model. As shown in Figure 6G, treatment with cysteamine or cystamine significantly reduced *Mtb* replication even more efficiently than the treatment with capreomycin in GLS, and the combined administration of capreomycin with the TG2 inhibitors did not provide any addictive effect. Conversely, the combined administration of amikacin with cystamine or cysteamine warranted an enhanced restriction of intracellular *Mtb* compared with the treatment with any of these drugs alone. Taken together, these results indicate that cystamine and cysteamine can synergize with a second-line anti-TB drug as amikacin, supporting their potential usefulness as HDTs for TB (Figures 7A,B).

## Discussion

Our recent report indicating that genetic and pharmacological inhibition of TG2 restricts *Mtb* replication in macrophages (16) prompted us to investigate the potential usefulness of the TG2 inhibitors cystamine and cysteamine as HDTs against TB. In this study, using a panel of *in vitro* experimental assays, we show that cysteamine and cystamine, two known inhibitors of TG2, can restrict *Mtb* replication in macrophages infected with the *Mtb* H37Rv reference strain and a panel of clinical isolates representative of different phylogeographic lineages. Interestingly, we analyzed the AIPD in THP-1 mRFP-GFP-LC3B cells infected with *Mtb* and observed, for the first time, that cystamine inhibited autophagy while restricting *Mtb* replication, confirming our previous observation (16). Overall, the results of this study support the potential usefulness of the TG2 inhibitors cysteamine and cystamine as HDTs against TB.

TG2 is known to contribute to a few important pathologies (15). Among the drugs that inhibit TG2, there are two small molecules, cystamine and cysteamine. These two drugs are safe when administered in humans; cysteamine is used to treat cystinosis (33, 34), and both cysteamine and cystamine have been used in human clinical trials in the treatment of diseases which directly or indirectly implicate TG2 and autophagy deregulation such as Huntington disease (35), cystic fibrosis (36, 37), and celiac disease (38). It is noteworthy that cystamine has been used as a support treatment in cancer therapy (39). In keeping with the data reported in this study, TG2 and autophagy are both up-regulated in cancer, playing a crucial role in oncogenesis (39, 40). Thus, the inhibitory action exerted by cysteamine and cystamine both on autophagy and TG2 could represent an efficient approach to favor the sensitization of cancer cells to chemo/radio/immune therapy. Our results showing that cysteamine and cystamine have an anti-TB activity when administered in a monocyte-derived macrophage cell line, in primary macrophages, or PBMCs infected with *Mtb* suggest that these drugs can be safely used as HDTs for TB (9).

Cystamine and cysteamine are reducing agents that can affect cell metabolism by increasing glutathione and L-cysteine level (35, 41). Recently, it has been demonstrated that L-cysteine or NAC can promote respiration in axenic *Mtb* culture, preventing the emergence of drug tolerance against the two most powerful anti-TB drugs, isoniazid and rifampicin (20). In these experiments, L-cysteine or NAC where administered at a concentration of 4 mM, which is five times higher than the concentration we used in our experiments involving *Mtb*-infected macrophages. Indeed NAC administered at a concentration of 10 mM was shown to directly decrease *Mtb* replication (42). However, in this study, we show a reproduction of the experimental conditions indicated in Vilcheze et al. (20), wherein cystamine or cysteamine, when administered at the concentrations used in *Mtb*-infected macrophages (400 and 800 μM, respectively), did not exert any direct activity against *Mtb* cultured in axenic media and did not prevent the emergence of drug tolerance against isoniazid. It follows that the anti-tuberculosis activity of cysteamine and cystamine that we observed in THP-1 monocyte-derived macrophages, primary macrophages, and PBMCs is not the result of a direct effect on *Mtb*. Moreover, Vilcheze et al. (20) showed that only the molecules with a free thiol group (as L-cysteine and NAC) may enhance *Mtb* metabolism, while the oxidized form, as cystine, does not exert any activity. Conversely, in our experiments, both cysteamine and cystamine similarly inhibit *Mtb* intracellularly in infected macrophages. This suggests that the mechanism of the anti-TB activity of the two anti-TG2 drugs described in the present study is different from that observed when L-cysteine or NAC is administered at a much higher concentration in *Mtb* axenic cultures.

It remains to be elucidated how the impairment of autophagy homeostasis by cysteamine and cystamine may contribute to restrict *Mtb* replication. It is well-established that induction of autophagy by various stimuli, such as rapamycin, IFN-γ, and Vitamin D3, promotes the lysosomal degradation of *Mtb* (43). However, the role of basal autophagy in infected macrophages appears to be more complex. We have recently demonstrated that *Mtb* strains from ancient and modern lineages have a different impact on the basal autophagy flux (25). While the ancient lineages impair the autophagic flux, infection with the modern strains leads to a stimulation of this process, which is dependent on the increased production of IL1-β triggered by these mycobacteria (25). This induction of autophagy is however ineffective in restricting *Mtb* growth but rather correlates with more exuberant *Mtb* replication, perhaps by sustaining its metabolic requirements in the infected cells (25, 44). These observations lend support to the hypothesis that a blanket inhibition of the autophagic flux in *Mtb*-infected macrophages may be detrimental for *Mtb*, perhaps because it would activate apoptosis, and that *Mtb* may manipulate this process in a more complex and dynamic way (45, 46). Autophagy is a complex and conserved process that involves multiple autophagy-associated enzymes; yet, apart from Atg5, autophagy-deficient mice do not show increased susceptibility to *Mtb* infection (47). Interestingly, the dramatic difference in the inflammatory response was the predominant driver for the enhanced susceptibility to *Mtb* infection in ATG5-deficient mice (47), highlighting the remarkable consequences that disruption of autophagic homeostasis can have during *Mtb* infection. These experimental observations suggest that *Mtb* has developed multiple strategies to escape autophagy engulfment and regulate the autophagy flux to fine-tune its pathogenetic strategies. Based also on the evidences generated in this study, we propose that impairment of the autophagic flux by the TG2 inhibitors is detrimental for *Mtb* intracellular growth. Further experiments are required to elucidate the functional link between impairment of autophagy homeostasis and *Mtb* growth and its consequences on inflammation when cells are treated with cysteamine and cystamine.

*Mycobacterium tuberculosis* complex (MTBC) is a genetically monomorphic species which evolved by clonal expansion since more than 100,000 years ago, leading to seven phylogeographic lineages which show different pathogenetic and virulence properties (27, 28). Most of the human TB cases at global level are caused by *Mtb* strains belonging to the modern lineages L2, L3, and L4, which seem to show enhanced pathogenic properties (29, 48, 49). More recent evidences indicate that even within these modern lineages, some clades or clusters may be more successful or virulent than others, indicating that the relative little genetic variability within *Mtb* can nevertheless have significant impact on infection outcome (28, 49). We and others have recently shown that *Mtb* strains of diverse lineages and clades can differently manipulate the autophagic process in infected macrophages, with consequences in terms of intracellular survival and cytokine/chemokine secretion (25, 50, 51). In this study, we show that the inhibition of TG2 by cysteamine or cystamine can effectively inhibit intracellular *Mtb* regardless of MTBC lineage. Indeed *Mtb* strains of the H3 and Beijing clade, which are characterized by an enhanced *in vitro* virulence compared to the other lineages (25), were inhibited by the anti-TG2 drugs, although at a lower level compared with the results observed with the other *Mtb* strains. Since *Mtb* H3 was shown to modulate the autophagy flux differently compared to the other *Mtb* strains, exploiting the autophagic process for its own survival (25), it is possible that the anti-TG2 drugs cysteamine and cystamine are less effective in inhibiting the intracellular *Mtb* H3. Nonetheless, these results demonstrate that cysteamine and cystamine have an antibacterial activity against several *Mtb* clinical strains representative of the global diversity of MTBC and support the finding that these anti-TG2 molecules are acting as HDTs by boosting macrophage antimicrobial responses.

Inhibition of TG2 and the ensuing effect on autophagy, in addition with the capability of these drugs to increase the generation of glutathione-S-transferase (52), may have consequences on the pattern of chemokines and cytokines secreted by infected macrophages. To evaluate the activity of cystamine and cysteamine in a more complex system, involving multiple cell types, we implemented the *ex vivo* model of GLS (31, 53). Treatment with cystamine or cysteamine of PBMCs infected with the *Mtb* H37Rv reference strain and the *Mtb* clinical strain H3 indicates a significant reduction in the total bacterial burden in GLS, although no major differences in GLS size were observed. These results indicate that the two anti-TG2 molecules can exert their anti-TB activity even in this *ex vivo* model of infection, further supporting their role as HDTs for TB.

HDTs against TB shall ideally serve to improve and eventually shorten current anti-TB regimens during treatment of drug-susceptible TB and, most importantly, drug-resistant TB. In fact, regimens against MDR-TB are longer and more toxic primarily because second-line drugs show reduced antimicrobial activity compared to isoniazid and rifampicin. Since the success rate for drug-susceptible TB is around 95% (54), we anticipate that any HDTs against TB will be tested and the activity will be measured in MDR-TB patients receiving second-line drugs. It is remarkable that cysteamine, and more robustly cystamine, can reduce intracellular *Mtb* growth similarly to the two aminoglycosides tested, underscoring on one side the potential antimicrobial activity of these two molecules and on the other the poor activity of the second-line drugs. Given the important potential clinical implications, we investigated the synergistic activity of the two anti-TG2 molecules with second-line drugs as those of the aminoglycoside class. The finding that cysteamine and cystamine synergized when administered in combination with capreomycin and most importantly with amikacin in primary human macrophages infected with *Mtb* and in the GLS *ex vivo* model of infection further highlights the potential usefulness of these two anti-TG2 inhibitors as HDTs against TB.

In conclusion, this study shows for the first time that cystamine and cysteamine display anti-*Mtb* activity while inhibiting host cell autophagy. These safe FDA-approved drugs have high potential applications against *Mtb* infection in combination with canonical anti-TB regimen to improve and shorten regimens against drug-susceptible TB and most importantly during treatment of MDR-TB patients or of patients which are at higher risk of non-compliance as migrants or homeless. In the future, specifically designed clinical trials should validate the efficacy for their utilization in the clinical practice, opening a new avenue in the treatment of TB.

## Materials and Methods

### Reagents and Bacterial Strains

The *M. tuberculosis* strain H37Rv, *Mtb* complex clinical strains (MTBC), and *M. bovis* BCG were isolated at the Fondazione Policlinico Gemelli IRCCS, Università Cattolica del Sacro Cuore (25, 55). The strains were grown in Middlebrook 7H9 (Difco, Sparks, MD) supplemented with 10% (vol/vol) oleic acid-albumin-dextrose-catalase (OADC; Difco), with 0.2% glycerol (Microbiol, Cagliari, Italy) and 0.05% Tween 80 (Sigma-Aldrich, St. Louis, MO) at 37°C. Mycobacterial cultures were harvested at late log phase, glycerol was added at 20% final concentration, and 1-ml aliquots stored at −80°C. All experiments with *Mtb* strains were carried out in biosafety laboratory level 3 (BSL-3), following standard safety procedures.

### Growth of *Mtb in vitro* Cultures

*Mtb* H37Rv cultures were diluted until a final concentration of ≈10^7^ CFU/ml, treated with the appropriate chemicals (cystamine 400 μM, cysteamine 800 μM, NAC 4 mM, INH 7.3 μM, or the combination INH/Cystamine, INH/cysteamine, INH/NAC using the same concentrations as the individual treatment), AND incubated at 37°C with shaking for the duration of the experiment, and CFU were obtained by plating serial dilutions. Plates were incubated at 37°C for up to 6 weeks. All experiments were carried out in BSL-3.

### Study Participants

The PBMCs were derived from healthy donors. Participants were recruited among people who had recently tested negative for QFT negative, not vaccinated with BCG, male, Caucasian, and aged between 30 and 35 years. Written informed consent was obtained from each donor.

### Cell Cultures

Human THP-1 cells *wt* were stably transduced with a retroviral vector encoding GFP-RFP-LC3 (56). *Wt* and transgenic THP-1 were grown in RPMI 1640 supplemented with glutamine (2 mM) and 10% FBS. Cells were treated with 20 nM PMA (Sigma-Aldrich, St. Louis, MO) for 24 h to induce their differentiation into macrophages, then washed three times with PBS, and maintained in 5% FCS.

Peripheral blood mononuclear cells (PBMCs) were obtained from healthy donors. PBMCs were isolated by density gradient centrifugation. Monocytes were purified from PBMCs by positive sorting, using anti-CD14–conjugated magnetic microbeads (Miltenyi Biotec, Auburn, CA). Human monocyte-derived macrophages (hMDM) were obtained by cultivating adherent monocytes for 5–6 days in X-Vivo 15 medium (Lonza, Walkersville, MD), 2% human serum (Euroclone, Paignton, United Kingdom) at 37°C in a 5% humidified atmosphere until macrophage differentiation (25).

Human cells were infected with different strains of MTBC [multiplicity of infection (MOI) 1: 1], and at various time-points (4 h, 2 and 7 days for hMDM), cells were washed twice with sterile phosphate-buffered saline (PBS) to remove extracellular bacteria, lysed in 0.01% Triton-X100 (Sigma-Aldrich, St. Louis, MO) and intracellular bacterial loads (in CFU) determined as previously described (57).

To assess the synergistic effect of cystamine and cysteamine with standard antibacterial drugs, we added 4 h and 3 days post-infection, respectively for hMDM instead of hMMO and GLS, capreomycin (4 μg/ml) (Sigma-Aldrich, St. Louis, MO), amikacin (1 μg/ml) (Sigma-Aldrich, St. Louis, MO) and combination of these drugs with cystamine and cysteamine.

### Granuloma-Like Structure Formation and Quantification

PBMCs were obtained from healthy donors and isolated as described above. PBMCs (containing ~1 × 10^5^ monocytes) were immediately infected with *Mtb* at a MOI of 1:1 and incubated for up to 10–12 days, during which time granuloma was developed and analyzed (31, 58). The analysis of stage of GLS has been done daily by using an inverted light microscope. At least 12 separate fields per sample were used to establish the area and total number of GLS (31). Intracellular bacterial growth was assessed by counting the CFU; infected GLS were lysed at different time-points (3, 6, and 12 days post-infection) as described previously (57).

### Confocal Microscopy

Images were obtained by using an inverted confocal microscope; the slides were then placed on the inverted confocal microscope (Nikon A1 MP) equipped with an on-stage incubator (*T* = 37°C, 5% CO_2_, OKOLAB), and 32 channel spectral images were obtained using a ×60 objective (NA 1.4) under 488-nm excitation for Nile Red. Internal photon multiplier tubes collected images in 16-bit, unsigned images at 0.25 ms dwell time. mRFP-GFP-LC3 was excited by an argon-ion laser line (excitation wavelength, 488 nm; emission ranges, 500–550, 570–620 nm). DsRED fluorescence was monitored in the channel 500–550 nm. Photomultiplier tube gain values were kept fixed during the experiment. Pinhole was set to 1 A.U.Z-. Analysis of images acquired was performed with ImageJ 1.41 (NIH). AIPD determination was obtained following Maulucci et al. (21). Briefly, the *R* index was obtained by calculating the ratio between fluorescence emissions in the 500–550 nm (*F*_G_) and 570–620 nm (*F*_R_) ranges, upon sample excitation at 488 nm. By mapping *R* over the entire microscope scanning field, *R* images can be created with the homemade downloadable software Redox Maps Generator Green (59), and red images were overlaid; maxima of red and green channels, representing autophagy intermediates (“puncta”), were retrieved by the FIND MAXIMA plugin (ImageJ). Regions of interests, including whole organelles, were manually drawn in correspondence of the maxima, and fluorescence intensity values were measured directly on the *R* image through the SYNC WINDOWS plug-in (ImageJ). Puncta without detectable EGFP fluorescence were minimized to <5% of the total number by setting adequate values for photomultipliers. At least 50 cells per sample were analyzed to build the histogram. Fluorescence intensities and intensity ratio data were presented as mean ± SD, and differences were assessed by using χ^2^-test. Values of *p* < 0.05 were considered as significant.

### Statistics

Data were analyzed using the GraphPad Prism software, version 7.02 for Windows (GraphPad Software, San Diego, CA). All experiments were performed at least three times in triplicate. Growth of *Mtb* H37Rv in *in vitro* cultures was evaluated using one-way ANOVA with Dunnett's multiple-comparisons test against *Mtb* H37Rv untreated; the statistical significance of the differences between MTBC strains was evaluated using two-way ANOVA with Dunnett's multiple-comparisons test against each strain with untreated condition. The healthy donors used for GLS formation were adult (18–45 years of age), uninfected, and non-vaccinated. Differences were considered significant if *p*-values were ≤0.05.

## Data Availability Statement

All datasets generated for this study are included in the article/Supplementary Material.

## Ethics Statement

The studies involving human participants were reviewed and approved by L. Spallanzani National Institute for Infectious Diseases-IRCCS (INMI) Ethical Committee (approval number: parere 4/2009, amendment of February 2018; approval number: parere 68/2018). The patients/participants provided their written informed consent to participate in this study. Healthy donors were prospectively enrolled from January 2017 until March 2019.

## Author Contributions

IP, GD, and MP conceived the study. IP designed and performed the experiments, analyzed data, interpreted results, prepared figures, and wrote manuscript. GD analyzed data, interpreted results, and took the lead in writing the manuscript. DG, MP, and MSan contributed to analyze the data. GM performed and analyzed all data with confocal microscopy. GM and MD interpreted results and prepared figures that associate the *Mtb* infection and autophagy omeostasis. FD and MSal maintained, cultivated and prepared different TB strains. FD and MSal collected samples and contributed to perform the experiments. LP, AR, and GF contributed reagents and analyzed the data. AR and GF produced the transfected cells RFP-LC3-GFP. MSan interpreted results. All authors critically reviewed the manuscript.

### Conflict of Interest

The authors declare that the research was conducted in the absence of any commercial or financial relationships that could be construed as a potential conflict of interest. The handling editor declared a shared affiliation, though no other collaboration, with one of the authors, MP.
