# Supplementary material for: Inhibition of Transglutaminase 2 as a Potential Host-Directed Therapy Against Mycobacterium tuberculosis
Source: Front Immunol. 2020 Jan 24;10:3042. doi: 10.3389/fimmu.2019.03042 (PMC6992558; doi:10.3389/fimmu.2019.03042)
Supplement: Supplementary file 1 [file Image_1.PDF]

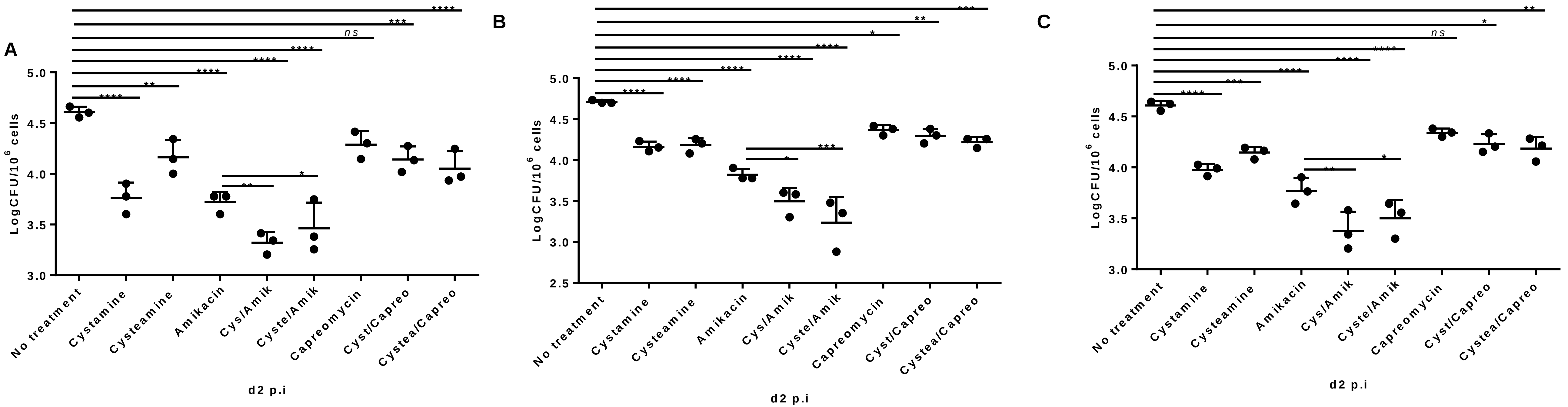

Supplementary Figure 1: Evaluation of the synergic effect of TG2 inhibitors with aminoglycosides in human primary monocyte-derive macrophages (hMDM) results from individuals donors. hMDM were infected with *Mtb* reference (*Mtb H37Rv*) at MOI 1:1. At 4 hours post-infection, cells were treated with different drugs (cystamine 400 µM; cysteamine 800 µM); capreomycin 4 µg/ml; amikacin 1 µg/ml; and the combination of the aminoglycosides with cystamine and cysteamine). At 2 days post-infection, cells were lysed to measure the number of viable bacteria by plating serial dilutions to determine CFUs. Scatter Dot Plot from three independent donors A-B-C. Statistical analysis was performed by two-way ANOVA followed by Turkey post-test (* $p<0.05$ ** $p<0.01$, *** $p< 0.005$, **** $p< 0.001$).
